# Acoustic Sensors Based on Amino-Functionalized Nanoparticles to Detect Volatile Organic Solvents

**DOI:** 10.3390/s17112624

**Published:** 2017-11-14

**Authors:** Daniel Matatagui, Oleg Kolokoltsev, José Manuel Saniger, Isabel Gràcia, María Jesús Fernández, Jose Luis Fontecha, María del Carmen Horrillo

**Affiliations:** 1 Centro de Ciencias Aplicadas y Dessarrollo Tecnológico (CCADET), UNAM, 04510 Ciudad de Mexico, Mexico; oleg.kolokoltsev@ccadet.unam.mx (O.K.); jose.saniger@ccadet.unam.mx (J.M.S.); 2 Instituto de Microelectrónica de Barcelona (IMB), CSIC, Campus UAB, 08193 Bellaterra, Spain; isabel.gracia@imb-cnm.csic.es; 3 Instituto de Tecnologías Físicas y de la Información (ITEFI), CSIC, Serrano 144, 28006 Madrid, Spain; m.j.fernandez@csic.es (M.J.F.); joseluis.fontecha@csic.es (J.L.F.)

**Keywords:** Love wave, surface acoustic wave, gas sensor, functionalized-nanoparticles, solvent, spin coating

## Abstract

Love-wave gas sensors based on surface functionalized iron oxide nanoparticles has been developed in this research. Amino-terminated iron oxide nanoparticles were deposited, by a spin coating technique, onto the surface of Love-wave sensors, as a very reproducible gas-sensing layer. The gases tested were organic solvents, such as butanol, isopropanol, toluene and xylene, for a wide and low concentration range, obtaining great responses, fast response times of a few minutes (the time at which the device produced a signal change equal to 90%), good reproducibilities, and different responses for each detected solvent. The estimated limits of detection obtained have been very low for each detected compound, about 1 ppm for butanol, 12 ppm for isopropanol, 3 ppm for toluene and 0.5 ppm for xylene. Therefore, it is demonstrated that this type of acoustic wave sensor, with surface amino-functionalized nanoparticles, is a good alternative to those ones functionalized with metal nanoparticles, which result very expensive sensors to achieve worse results.

## 1. Introduction

Volatile organic solvents are commonly found in the environment, indoor air, and workplaces and can be present in a large variety of consumer products, such as surface cleaning formulations, varnishes, inks and adhesives, cosmetics, fuels, etc. [1]. The main risk of intoxication generally occurs in the workplace, where substances that contain these solvents are manipulated for long periods of time. Aromatic hydrocarbons such as toluene and xylene are particularly alarming ones due to their carcinogenic nature [2], even at very low concentrations (ppb), as long-term exposure to aromatic hydrocarbons may result in lymphatic and hematopoietic cancers [3] and their inhalation severely affects nervous system and blood production processes [3,4]. An important group of solvents that produce side effects are alcohols, for instance butanol is related to central nervous system depression, and also causes severe eye irritation and moderate skin irritation [5,6] or isopropanol that at low concentrations causes irritation of the respiratory system, eye, and mucous membranes, however at high concentrations it also causes central nervous system effects such as dizziness, nausea, hypotension, and hypothermia [7]. According to the Occupational Health and Safety Administration (OHSA) , the time weighted average (TWA) 8-h limit (the concentration of a substance in air which may not be exceeded over a normal 8 h work period) for the chosen solvents are: 15 ppm for butanol, 200 ppm for isopropanol, 20 ppm for toluene, and 100 ppm for xylene.

Due to the adverse effects of volatile organic solvents on humans, there has been an increasing need for developing new sensitive, low-cost and portable systems, which can respond in real time, for monitoring trace levels of volatiles in various environmental and industrial applications. Currently, there are some promising low-cost devices, with high sensitivity and low dimensionality known as chemical sensors, which are based on capacitive effects [8,9], resistive effects [10,11], optical fibers [12,13], field effect transistors (FETs) [14,15], surface acoustic waves (SAWs) [16,17] and quartz crystal microbalances (QCM) [18,19]. Among the SAW sensors, the Love-wave devices have generated great interest as gas sensors due to their potential to confine the wave energy in a thin guiding layer, provided that the velocity of the acoustic wave in the guiding layer is slower than that in the substrate, obtaining in this way a very high mass sensitivity. Commonly, a thin sensitive layer on the top of the structure can be used as an active medium that interacts with the environment but the energy is not confined in this layer [20]. In addition, in recent years, many of the sensors developed for specific applications in toxic chemical agent sensing, are based on nanomaterials. Nanostructured materials improve the sensitivity and velocity of the sensitive layer response due to their large ratio of surface area respect to their volume. Particularly, layers of nanoparticles are being used in SAW gas sensors and their high sensitivity to the gases has been demonstrated, although there are still few studies on this topic [21,22]. Moreover, nanoparticle surface can be improved by incorporating organic groups to its nanostructure, in order to obtain a higher affinity with defined gases. In this work surface-functionalized nanoparticles are proposed as high efficiency sensing layers for a Love-wave sensor, choosing amino-functionalized iron oxide nanoparticles because the amino group has a high reactivity, is a rich source of chemistry and can react with many chemicals with different intensity [23,24,25,26]. Different solvents used in the industry have been measured due to the good results obtained sensing N,N-dimethylformamide [26].

## 2. Materials and Methods 

### 2.1. Device Design 

**Figure 1:**
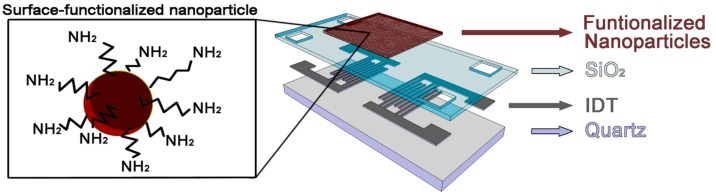
A 3D scheme representing a Love wave sensor with a functionalized nanoparticle sensitive layer. IDT: interdigital transducer.

The SAW device used in this work was based on a shear horizontal surface acoustic wave (SH-SAW) propagated on a piezoelectric material. In our case the piezoelectric was a ST-cut quartz substrate and the propagation direction of the wave was perpendicular to the x crystallographic axis. This SH-SAW, with a wavelength (λ) of 28 μm, was generated and detected by interdigital transducers (IDTs), which transform the radiofrequency (RF) signals in mechanical waves by means of the piezoelectricity of the substrate. The IDTs were made using standard lithographic techniques, depositing an aluminium layer with a thickness of 200 nm through RF sputtering and forming a delay line (DL). A double finger electrode was designed to obtain destructive interference of the wave reflection that occurs in the IDT, repeating this structure 75 times to form each IDT. The spacing, center to center between IDTs (Lcc) was 150 λ and the acoustic aperture (W) was 75 λ. Finally, the SH-SAW is guided in a film of SiO_2_ deposited by PECVD in order to obtain a Love-wave. The thickness of SiO_2_ was of about 3.5 μm, and the synchronous frequency 164 MHz. Finally, a 4 mm × 9 mm × 0.5 mm device is obtained (Figure 1).

### 2.2. Sensitive Layer

Iron oxide nanoparticles (6.5 nm ± 3.0 nm) functionalized with oleic acid and 11-amino-undecanoic acid, resulting in amino-terminated iron oxide nanoparticles, dispersed in water 25–35% wt, were provided by Sigma-Aldrich (Cat. 51,238, Cd. De Mexico, Mexico. Then the functionalized nanoparticles were deposited onto SiO_2_ guiding layer by spin coating technique with a very good adherence, at a rate of 4500 rpm during 1 min, obtaining, in this way, the sensing layer. Therefore, the water was removed and the adherence of the functionalized nanoparticle layer on the SiO_2_ surface was increased.

### 2.3. Experimental Setup

**Figure 2:**
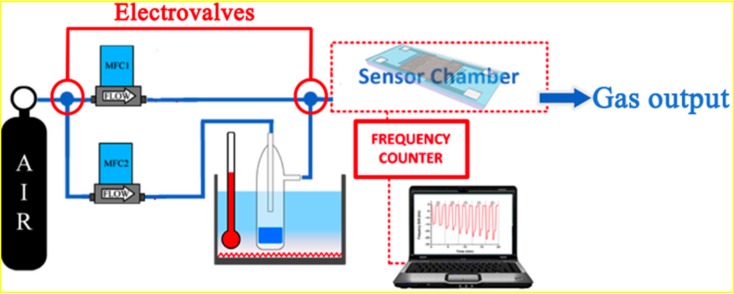
Experimental setup used to measure different concentrations of solvents in real time.

The concentrations of the different solvents, butanol, isopropanol, toluene and xylene were calculated using Antoine’s Equation [27]. The volatiles were diluted in synthetic air, controlled by a mass flow controller in order to achieve the desired concentration. The volume of each sample was 5 mL. It was kept at a constant temperature (4 °C) in a thermal bath for 30 min (headspace time) before being carried to the sensor chamber. Air flow in the chamber was 100 mL/min and the exposure and purge times were 5 min. The Love-wave sensor was integrated in an oscillator circuit that leads the oscillation with a specific frequency, which was used as output signal and read by a frequency counter. The experiment control and data acquisition in real time were implemented with a PC by means of a custom software. The experimental setup is shown in Figure 2.

## 3. Results

### 3.1. Electrical Characterization

**Figure 3:**
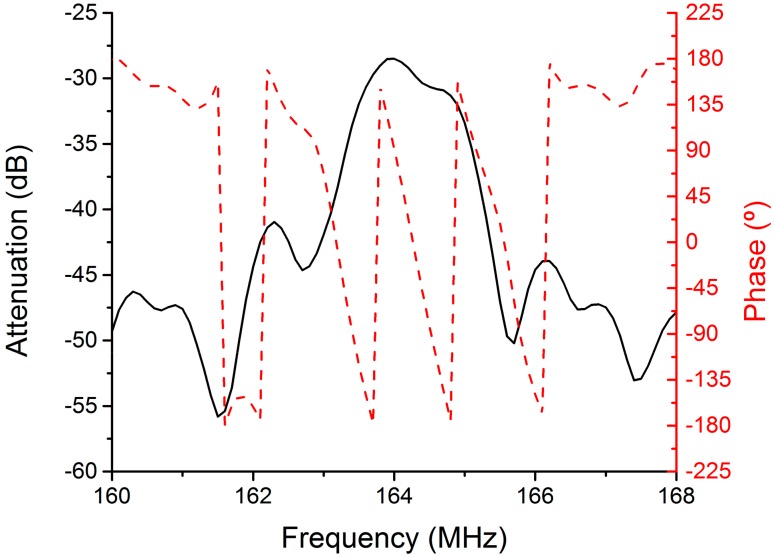
Amplitude and phase of transmission S_21_ of a Love-wave sensor coated with an amine-functionalized iron oxide nanoparticle sensitive layer.

A 360B Automatic Network Analyzer (Wiltron), was used to characterize the Love-wave device coated with the functionalized iron oxide nanoparticles in RF range, where acoustic waves were propagated. The frequency response was recorded (Figure 3), resulting in an acoustic propagation wave centered about 164 MHz with a minimum attenuation (about 28 dB) of the transmission S_21_. In addition, a linear phase corresponding to principal acoustic propagation was observed, meaning that there was no parasitic interference in the signal transmission, getting a stable oscillation when the device was introduced into the amplifier loop.

### 3.2. Gas Characterization

The sensor was tested with different concentrations of the solvents butanol (25, 50, 75, 100, 150 ppm), isopropanol (400, 600, 800, 1000, 1200 ppm) toluene (200, 400, 600, 800, 1000 ppm), xylene (50, 100, 150, 200 ppm). These values were the lowest concentrations that could be achieved with the gas generator available in this experiment. The surface-functionalized nanoparticle layer interacted with the chemical solvent and consequently the wave propagation velocity decreased by the amount of mass incorporated on the surface, and in this way, the frequency of the Love-wave DL was used as controller of an oscillating device.

**Figure 4:**
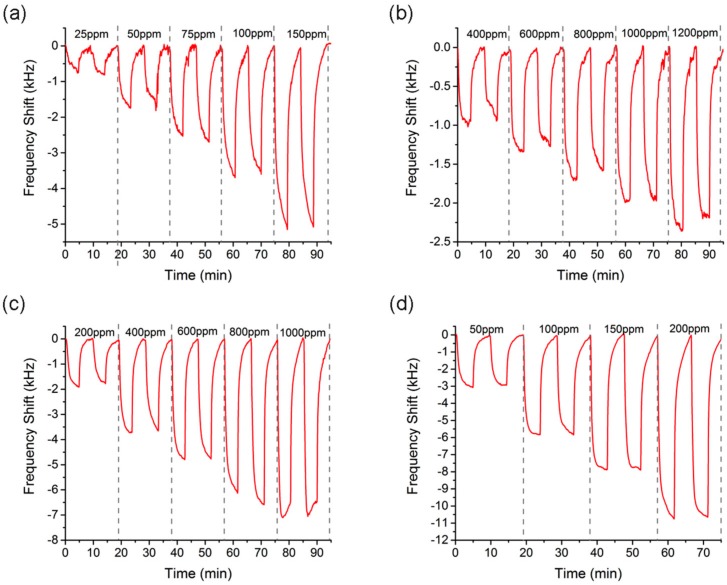
Real time response of the Love-wave sensor for different concentrations of the solvents: (**a**) butanol, (**b**) isopropanol, (**c**) toluene and (**d**) xylene.

Each concentration was measured twice, obtaining high and repetitive responses of the sensor. In addition, different concentrations were detected, showing higher sensor responses for increasing concentration steps. Exposure and desorption times were fixed at 5 min for each solvent (Figure 4a–d).

In general, the responses for each solvent were very high and reproducible (Table 1), and besides these ones were higher than the responses given by this same type of sensors with sensitive layers doped with metal nanoparticles [22]. These good results are attributable to two sorption mechanisms that take place when the sensitive layer interacts with the solvents: (1) the amino groups react with the organic groups of the solvent according to their affinity, because of the N-H bond is easily broken (chemisorption). (2) The solvent molecules are physisorbed on the iron oxide nanoparticles, due to the great surface area of the same ones. Therefore, the sensitivity and selectivity are incremented for each solvent as the functionalization of nanoparticle layer is made with amino groups [23].

**Figure 5:**
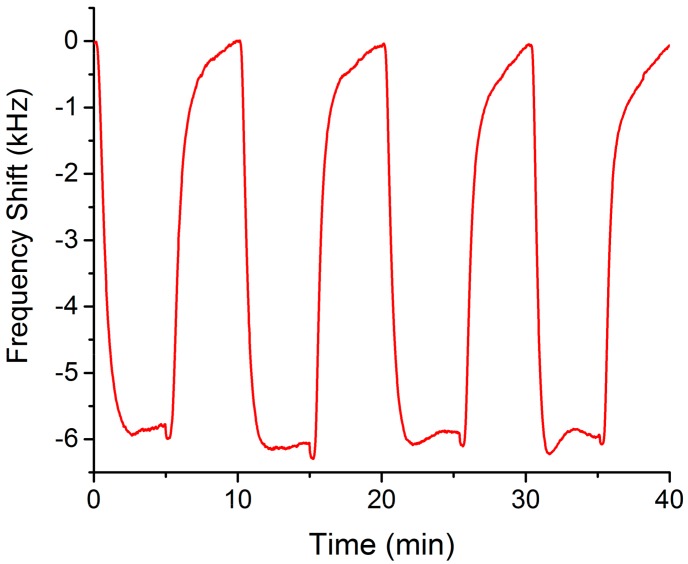
Real time response of the Love-wave sensor for four repetitions of 100 ppm of xylene, as a control measurement.

In the preset study a control test was carried out measuring two repetitions of 100 ppm of xylene (Figure 4) with a response average of 5840 Hz and a coefficient of variance of 2%. Six months before, xylene had been measured through four repetitions (Figure 5), in this case, the average of the response measurements was 5930 Hz. Therefore, the sensor showed long-term repeatability and reproducibility.

**Figure 6:**
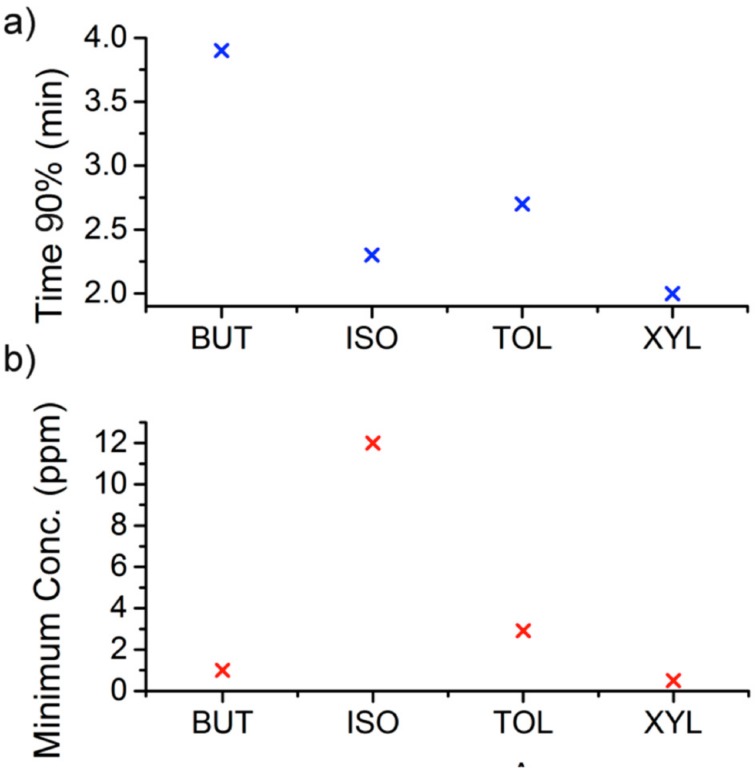
For the tested solvents: (**a**) Signal variation equal to 90% of the complete magnitude of sensor signal; (**b**) Detection limit obtained from minimum concentrations measured.

On the other hand, the lowest concentration measurements of the volatile organic solvents were considered to estimate the response time of sensor. The response time of sensor was defined as the time at which the device produced a signal change equal to 90% (τ_90_) of the complete magnitude of sensor signal (Figure 6a).

We can point out that the response kinetics were fast, since the response times of the sensor to the lowest concentrations for the different tested volatile solvents were 3.9 min for butanol, 2.3 min for isopropanol, 2.7 min for toluene and 2 min for xylene, giving thereby an alarm signal before the side effects of these organic solvents started.

The detection limits were calculated, taking into account that the noise of the oscillator was below 10 Hz and the minimum detectable change was considered 30 Hz, being about 1 ppm for butanol, 12 ppm for isopropanol, 3 ppm for toluene and 0.5 ppm for xylene, which were widely lower that TWA 8-h limit for each solvent (Figure 6b).

**Figure 7:**
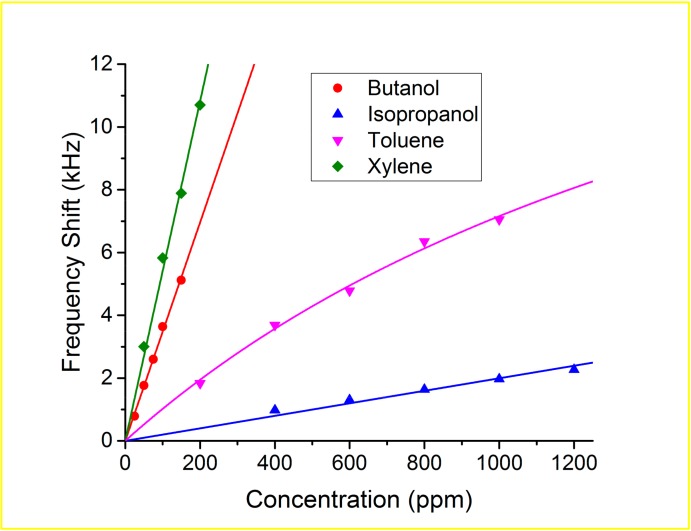
Response of the Love sensor for different solvents.

The sensor had a good linear correlation between the frequency shift and concentration in the case of butanol, isopropanol and xylene, however in the case of the toluene a Langmuir isotherm fit was more suitable [28], as it is shown in Figure 7.

## 4. Conclusions

The use of a low cost, easy, fast and repetitive technique (spin coating) to deposit functionalized nanoparticles as the sensitive layer of Love-wave sensors is presented in this work. Different advantages can be achieved for this type of sensors using functionalized nanoparticles, since the nanoparticles increase the sensing response, with regard to a continuous layer, due to their high surface area. In addition, the response to different solvents of the Love-wave sensor can also be controlled and improved by functionalization of nanoparticles with a particular chemical group. In our case, the amino groups on the surface of the iron oxide nanoparticles react with the organic solvents, due to their great reactivity, and of this mode the sensitivity and selectivity are incremented with respect to when the sensitive layer is only formed by nanoparticles. All the solvents tested were detected, to low concentrations, by the newly fabricated Love-sensor, with different responses for each solvent. The detection limits of the sensor for the different volatiles was estimated at 1 ppm for butanol, 12 ppm for isopropanol, 2 ppm for toluene and 0.5 ppm for xylene, which were lower than the TWA 8-h limit.

In conclusion, the amino-functionalized Love-wave sensor shows good linearity, stability, reversibility, accuracy and very high and fast response for detecting volatile solvents at room temperature, improving upon the results obtained with metal-functionalized nanoparticles. Besides, it is important to highlight the low cost of amino-functionalized nanoparticles in comparison to metal-functionalized nanoparticles. We believe that this work opens up a promising field of research for e-noses based on Love-wave sensors, since it will be possible to fabricate Love-wave sensor arrays with sensitive layers of functionalized nanoparticles with appropriate chemical groups, which will have a high sensitivity and selectivity, allowing to detect, discriminate, and classify different volatile compounds, fact very important for the industrial and environmental applications.

## Figures and Tables

**Table 1:** Responses to solvents.

Butanol		Isopropanol		Toluene		Xylene	
Conc. (ppm)	Response (kHz)	Conc. (ppm)	Response (kHz)	Conc. (ppm)	Response (kHz)	Conc. (ppm)	Response (kHz)
25	0.79	400	0.98	200	1.84	50	3.00
50	1.76	600	1.31	400	3.69	100	5.83
75	2.60	800	1.64	600	4.78	150	7.89
100	3.64	1000	1.97	800	6.35	200	10.70
150	5.12	1200	2.27	1000	7.05		
